# Atrophy patterns in hippocampus and amygdala subregions of depressed patients with Parkinson's disease

**DOI:** 10.1007/s11682-023-00844-9

**Published:** 2024-01-03

**Authors:** Mingrui Qu, Bingbing Gao, Yuhan Jiang, Yuan Li, Chenhui Pei, Lizhi Xie, Yukun Zhang, Qingwei Song, Yanwei Miao

**Affiliations:** 1 Department of Radiology, The First Affiliated Hospital of Dalian Medical University, No. 222 Zhongshan Road, Xigang District, Dalian, 116011 China; 2 Department of Neurology, The First Affiliated Hospital of Dalian Medical University, Dalian, China; 3 GE Healthcare, Shanghai, China

**Keywords:** Parkinson’s disease, Depression, Amygdala, Hippocampus, Subregions, Magnetic resonance

## Abstract

We aimed to explore the subregional atrophy patterns of the amygdala and hippocampus in Parkinson's disease (PD) with depression and their correlation with the severity of the depressive symptom. MRI scans were obtained for 34 depressed PD patients (DPD), 22 nondepressed PD patients (NDPD), and 28 healthy controls (HC). Amygdala and hippocampal subregions were automatically segmented, and the intergroup volume difference was compared. The relationships between the volumes of the subregions and depression severity were investigated. Logistic analysis and Receiver operator characteristic curve were used to find independent predictors of DPD. Compared with the HC group, atrophy of the bilateral lateral nucleus, left accessory basal nucleus, right cortical nucleus, right central nucleus, and right medial nucleus subregions of the amygdala were visible in the DPD group, while the right lateral nucleus subregion of the amygdala was smaller in the DPD group than in the NDPD group. The DPD group showed significant atrophy in the left molecular layer, left GC-DG, left CA3, and left CA4 subregions compared with the HC group for hippocampal subregion volumes. Also, the right lateral nuclei volume and disease duration were independent predictors of DPD. To sum up, DPD patients showed atrophy in multiple amygdala subregions and left asymmetric hippocampal subregions. The decreased amygdala and hippocampal subregion volumes were correlated with the severity of depressive symptoms. The volume of right lateral nuclei and disease duration could be used as a biomarker to detect DPD.

**Supplementary Information:**

The online version contains supplementary material available at 10.1007/s11682-023-00844-9.

## Introduction

Parkinson's disease (PD) is a progressive neurodegenerative disorder. One of the most common non-motor symptoms, depression, affects up to 40% of patients with PD (Dooneief et al., 1992). However, because of the overlapping clinical symptoms, the depressive symptoms in PD are frequently unrecognized and subsequently underdiagnosed, thus often remaining untreated (Shulman et al., 2002).

The characteristic pathological biomarkers of PD are deposits of phosphorylated α-synuclein in Lewy bodies (LB) and dystrophic neurites (Jellinger, 2012). LB accumulation in specific brain areas of PD patients may damage emotion-related circuits (Huang et al., 2015). Among these areas, the amygdala seems to be the affected first. Overexpression of α-synuclein in the hippocampus triggers depressive-like behaviors, leading to synapse loss and microglia-mediated inflammation (Du et al., 2021).

Neuroradiology studies focused on brain structural and functional change have been used to further elucidate the potential mechanisms of depressed PD patients (DPD). Previous MRI studies have found that DPD was related to decreased gray matter volume in the parahippocampal gyrus, medial and anterior cingular cortex, orbitofrontal gyrus, and medial temporal gyrus (Feldmann et al., 2008). Depression was also associated with cortical thinning in limbic brain regions (Hanganu et al., 2017). Some studies found that depression scores are negatively correlated with hippocampal and amygdala volumes in PD patients (Goto et al., 2018; van Mierlo et al., 2015). Other studies in DPD patients reported atrophy in the bilateral amygdala, the main region related to emotion (Chagas et al., 2017; Surdhar et al., 2012). In addition, Vriend et al., demonstrated that anxiety symptoms in PD patients are negatively correlated with the volume of the left amygdala (Vriend et al., 2016). A functional MRI study on PD found that abnormal connectivity between the amygdala and hippocampus was related to depression (Lin et al., 2020).

The amygdala and hippocampus are composed of interconnected substructures. These substructures show different responses to pathological mechanisms and have diverse functions during emotional processing (Alarcón et al., 2015; Goossens et al., 2009). Previous studies on patients with major depressive disorder have shown that the main hippocampal body was relatively intact, while deformations localized to the subiculum and CA1 subregion extended into the CA2-3 subregions (Cole et al., 2010). A recent study found that atrophy and expansion effects in subregions of the hippocampus and amygdala exist simultaneously (Yao et al., 2020). Together, the above studies suggest that substructural alterations may have significant implications for elucidating the pathophysiology of depression. To better understand the subtle functional role of limbic substructures in DPD, this study investigated the amygdala and hippocampal subregion changes in DPD patients and their relationship with the severity of depressive symptoms, assisting in finding new diagnostic markers for DPD.

## Materials and methods

### Subjects

This study enrolled 56 PD patients, including 34 DPD and 22 nondepressed PD patients (NDPD), hospitalized in our hospital between February 2017 and May 2021. The inclusion criterion for PD was the fulfilment of the 2015 Movement Disorders Society (MDS) criteria for PD diagnosis (Postuma et al., 2015). The exclusion criteria for PD patients included: (1) other neurological diseases such as vascular Parkinsonism and Parkinsonism-Plus syndrome; (2) history of severe cranial organic lesions, head trauma, and neurological surgery; (3) history of alcoholism and drug abuse; (4) Dementia or apparent cognitive impairments, the score of Mini-Mental State Examination (MMSE), weighted by Chinese education, < 17 for illiterate subjects, < 20 for grade-school literate, and < 24 for junior high school and higher education literate. 28 age-and sex-matched healthy controls (HC) were recruited without severe psychiatric disorders such as anxiety and depression.

### Neuropsychological assessment

The neuropsychological assessment of all subjects was estimated by an experienced neurologist. The severity of the disease was quantified with the Hoehn and Yahr scale (H&Y). The severity of depressive symptoms was assessed using the Hamilton Depression Scale (HAMD), where those with HAMD score > 7 were assigned to the DPD group, and HAMD score ≤ 7 belonged to the NDPD group (Liao et al., 2021). The severity of anxiety symptoms was assessed using the Hamilton Anxiety Scale (HAMA). Finally, the cognitive state of subjects was estimated based on MMSE.

### MRI acquisition

All MR Imaging data were obtained on a 3.0 T GE Signa HDXT scanner equipped with an 8-channel head coil. A sagittal 3D magnetization-prepared rapid-acquisition gradient-echo T1-weighted sequence with the following parameters was collected: repetition time = 10.2 ms, echo time (shortest) = 4.2 ms, slice thickness 1.0 mm, flip angle = 13°, FOV = 256 × 256 mm^2^, matrix size = 256 × 256, voxel size = 1.0 × 1.0 × 1.0 mm^3^.

### Data processing

**Fig. 1:**
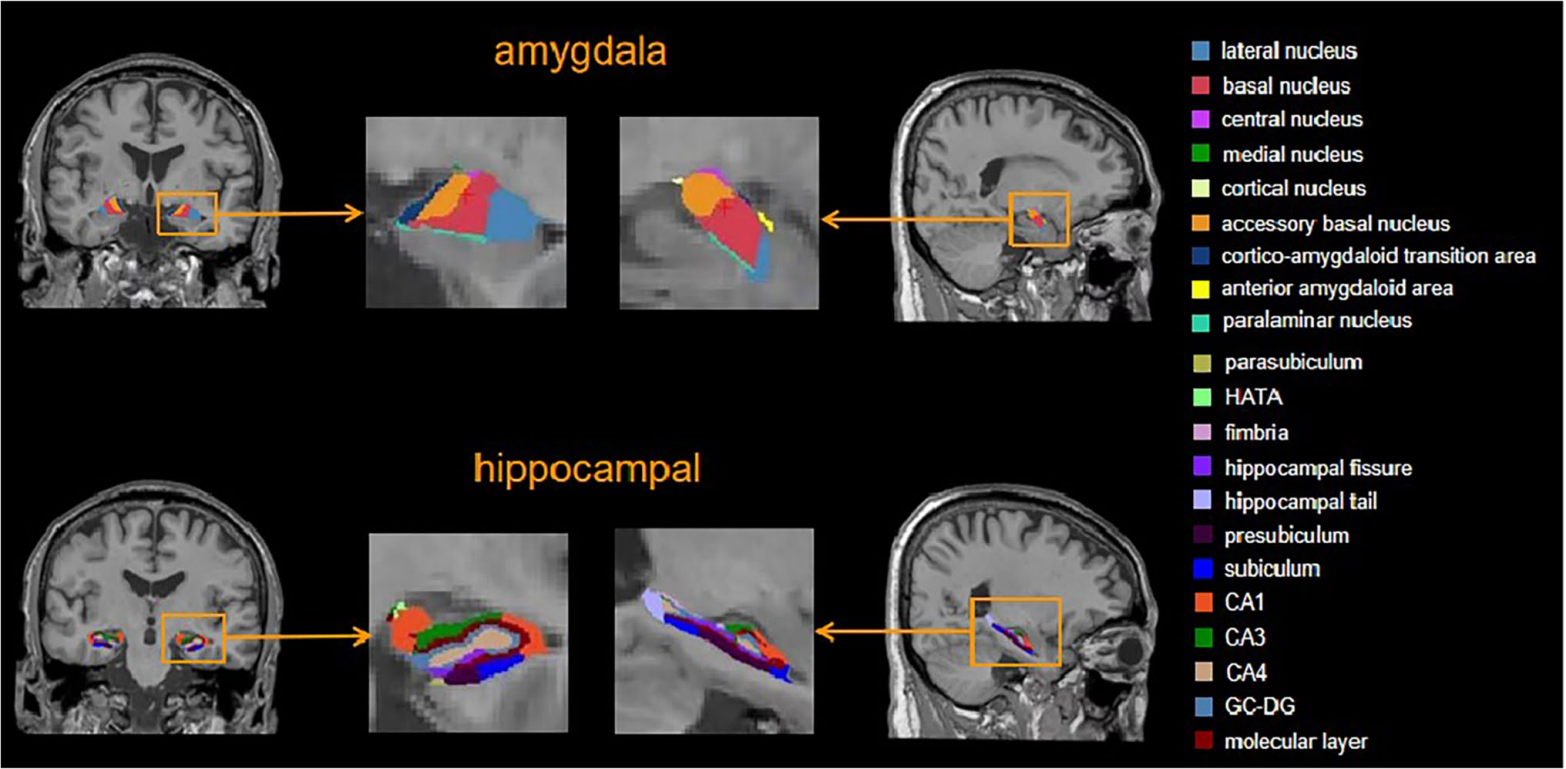
Coronal and sagittal 3D-T1-weighted images from the automated segmentation of Freesurfer hippocampal and amygdala subregions segmentation from a healthy individual. Abbreviations: GC-DG, Granule cell layer of the dentate gyrus; HATA, Hippocampus-amygdala transition area

3D T1-weighted image data were processed with the FreeSurfer 6.0 software (https://www.freesur fer.net/). The amygdala and hippocampal subregions were automatically segmented, and the volumes of bilateral amygdala and hippocampal subregions and estimated total intracranial volume (eTIV) were calculated. Detailed information on the technical aspects of this procedure has been described in previous reports (Iglesias et al., 2015; Saygin et al., 2017). The amygdala was divided into 9 following subregions: lateral nucleus, basal nucleus, accessory basal nucleus, anterior amygdaloid area, central nucleus, medial nucleus, cortical nucleus, cortico-amygdaloid transition area, and paralaminar nucleus. The hippocampus was divided into 12 following subregions: parasubiculum, presubiculum, subiculum, CA1, CA3, CA4, granule cell layer of the dentate gyrus (GC-DG), the hippocampus–amygdala transition area (HATA), fimbria, molecular layer, hippocampal fissure, and hippocampal tail (Fig. 1). All FreeSurfer outputs were visually inspected for quality and segmentation accuracy during the analysis. Since no apparent instances of incorrect segmentation were found, no subjects were excluded.

### Statistical analysis

Statistical analyses were performed using SPSS 26.0 software. The Shapiro–Wilk test was used to test the normality of data distribution. Chi-square test was performed for proportions. The age, education, MMSE, HAMD, and HAMA scores were compared using ANOVA among the three groups. Two-sample *t*-test was used to assess the differences in disease duration between the DPD and NDPD groups. H&Y stage was compared using Mann–Whitney *U* test. Correlation analysis to evaluate the correlation of HAMD scores with HAMA scores. ANCOVA was applied to compare the differences between the three groups' amygdala and hippocampal subregion volume. The False-Discovery Rate (FDR) method was used to correct for multiple comparisons. Partial correlation analysis was performed to evaluate the correlation of significantly reduced amygdala and hippocampal subfield volumes with the severity of depressive symptoms and disease duration (FDR corrected). Finally, a forward stepwise Logistic analysis was carried out. Statistically significant substructure volumes and clinical indexes were included in the model to identify independent predictors of DPD. The receiver operator characteristic curve (ROC) was used to evaluate the prediction model's performance. All analyses were adjusted for age, sex, education, and eTIV. *P* < 0.05 indicated statistical significance.

## Results

### Demographic and clinical data

The three groups had similar gender, age, education, and MMSE results (*P* > 0.05). DPD group had significantly higher disease duration and H&Y stage than NDPD group (*P* < 0.05). HAMD and HAMA scores were significantly higher in DPD group than NDPD and HC groups (*P* < 0.001). The demographic information and clinical data are summarized in Table 1. A moderate correlation between HAMD scores and HAMA scores in all PD patients (NDPD and DPD) (*r* = 0.646, *P* < 0.001). However, there is no correlation between HAMD scores and HAMA scores in the DPD or NDPD group (*P* > 0.05), separately.

**Table 1:** Demographic and clinical characteristics

Characteristics	DPD (*n* = 34)	NDPD (*n* = 22)	HC (*n* = 28)	χ^2^/*F*/*t/Z*	*P* value
Age (years)	63.53 ± 7.39 (40–79)	60.64 ± 5.46 (48–71)	60.25 ± 6.60 (50–71)	2.22^②^	0.115
Gender (male/female)	13/21	14/8	14/14	3.47^①^	0.176
Education (years)	10.44 ± 3.03 (6–16)	10.82 ± 2.95 (6–16)	11.32 ± 3.95 (4–18)	0.53^②^	0.590
Disease duration (years)	6.43 ± 6.05 (0.5–30)	2.85 ± 2.51 (0.3–12)	-	2.62^③^	**0.011**
H&Y stage (in %)			-	-2.04^④^	**0.042**
1	5(15%)	9(41%)			
1.5	2(6%)	-			
2	16(47%)	10(45%)			
2.5	5(15%)	1(5%)			
3	6(18%)	2(9%)			
MMSE	26.47 ± 1.79 (24–30)	26.81 ± 1.94 (24–30)	26.29 ± 2.62 (21–30)	0.39^②^	0.681
HAMD	13.85 ± 3.03 (9–20)	5.09 ± 1.41 (2–7)	4.79 ± 1.71 (2–7)	154.81^②^	** < 0.001**
HAMA	15.15 ± 3.94 (8–21)	7.27 ± 3.12 (1–13)	3.39 ± 2.56 (0–8)	101.03^②^	** < 0.001**

^①^ Chi-Squared test, ^②^ Analysis of variance (ANOVA), ^③^ Two-sample *t*-test, ^④^Mann–Whitney *U* test

*P* < 0.05 had statistical significance. Bold values indicate statistically significant *P* values. Abbreviations: *DPD* Depressed PD patients; *NDPD* Nondepressed PD patients; *HC* Healthy controls; *H&Y* Hoehn and Yahr stage; *MMSE* Mini-mental state examination; *HAMD* Hamilton depression scale; *HAMA* Hamilton anxiety scale

### Amygdala subregion volume differences among DPD, NDPD, and HC groups

**Fig. 2:**
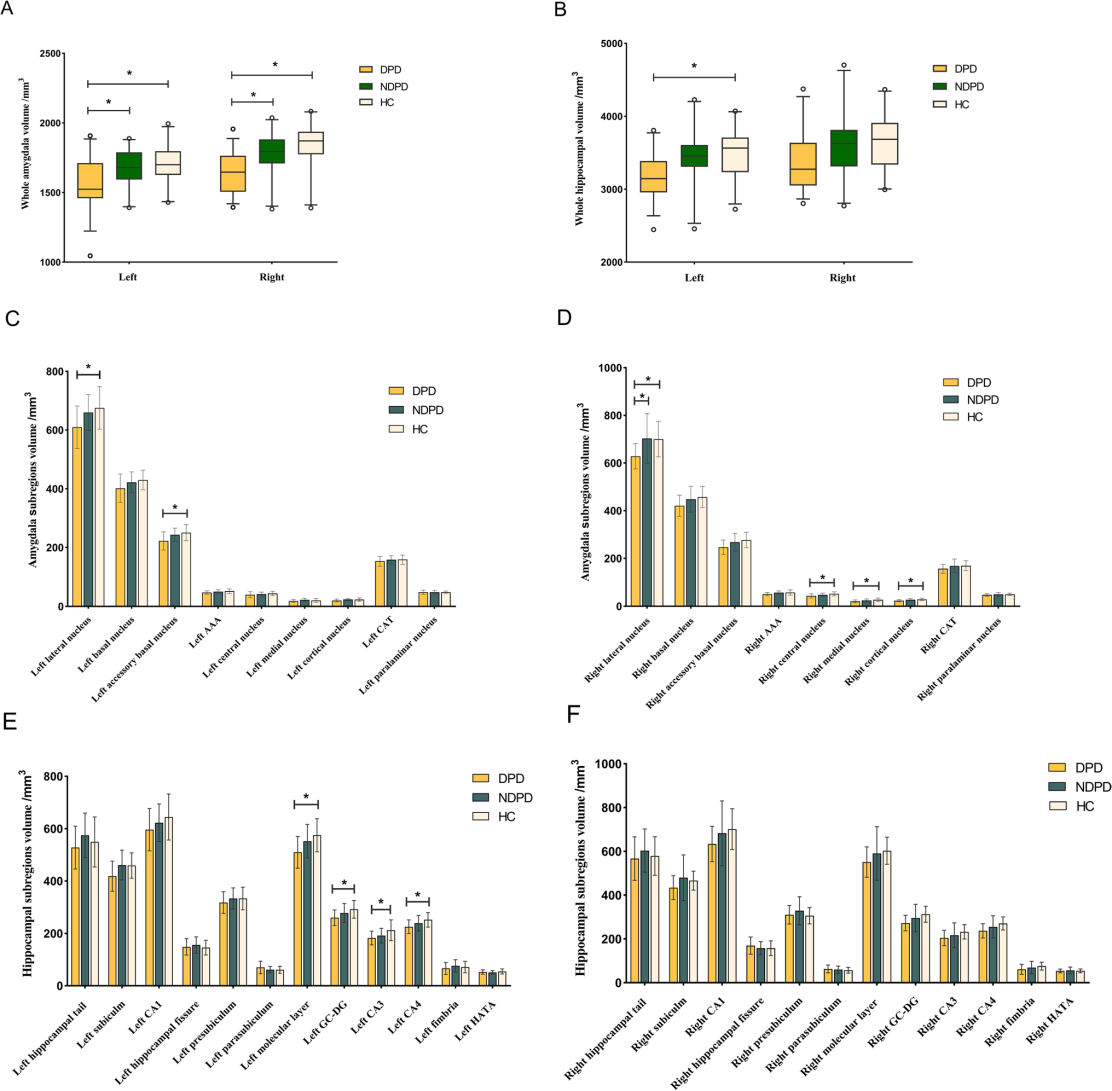
**A-B** Comparison of the global volume of bilateral amygdala and hippocampus among the DPD, NDPD, and HC groups. **C-D** Comparison of the volumes of bilateral amygdala subregions among the DPD, NDPD, and HC groups. **E–F** Comparison of the volumes of bilateral hippocampal subregions among the DPD, NDPD, and HC groups. Adjusted age, sex, education, and eTIV. ^*^ FDR corrected *P* value < 0.05. Abbreviations: DPD, Depressed PD patients; NDPD, Nondepressed PD patients; HC, Healthy controls; AAA, Anterior amygdaloid area; CAT, Cortico-amygdaloid transition area; GC-DG, Granule cell layer of the dentate gyrus; HATA, Hippocampus–Amygdala transition area

As shown in Fig. 2A, bilateral global amygdala volumes were significantly lower in DPD group than in NDPD group (left *P* = 0.033 and right *P* = 0.031) and HC group (left *P* = 0.026 and right *P* = 0.017). Further comparison of bilateral amygdala subregion volumes revealed significantly lower volumes in the bilateral lateral nucleus, left accessory basal nucleus, right cortical nucleus, right central nucleus, and right medial nucleus among the three groups (all *P* < 0.05). Post-hoc analyses showed significantly lower volumes in the DPD group than HC group for the bilateral lateral nuclei (left *P* = 0.016, right *P* = 0.019), left accessory basal nucleus (*P* = 0.012), right cortical nucleus (*P* = 0.004), right central nucleus (*P* = 0.004), and right medial nucleus (*P* = 0.007). However, compared with NDPD group, DPD group showed a significantly lower volume in the right lateral nucleus (*P* = 0.009) (Fig. 2C-D, Supplementary Table 1). We further controlled for the confounding interference of disease duration and H&Y stage and found that the right lateral nucleus (*P* = 0.022) and bilateral global amygdala volumes (*P* < 0.05) remained statistically significant between the DPD and NDPD groups. There were no statistically significant differences in the amygdala subregion volumes between the NDPD and HC groups (*P* > 0.05).

### Hippocampal subregion volume differences among DPD, NDPD, and HC groups

DPD group's left global hippocampal volume was significantly reduced than in HC group (*P* = 0.013) (Fig. 2B). Further comparison of hippocampal subregions volumes revealed significantly lower volumes in the left molecular layer, left GC-DG, left CA3, and left CA4 among the three groups (all *P* < 0.05). Any differences in the right hippocampal subregion volumes were no longer significant after FDR multiple comparison corrections. Post-hoc analyses of these four hippocampal subregions on the left showed differences between the DPD group and HC group in the left molecular layer (*P* = 0.004), left GC-DG (*P* = 0.003), left CA3 (*P* = 0.007), and left CA4 (*P* = 0.005) subregion volumes (Fig. 2E-F, Supplementary Table 2). There was no significant difference in hippocampal subregion volumes between the NDPD group and HC groups or between the two PD groups (*P* > 0.05).

### Correlation analysis of amygdala and hippocampus subregion volumes with HAMD scores and disease duration in DPD group

**Fig. 3:**
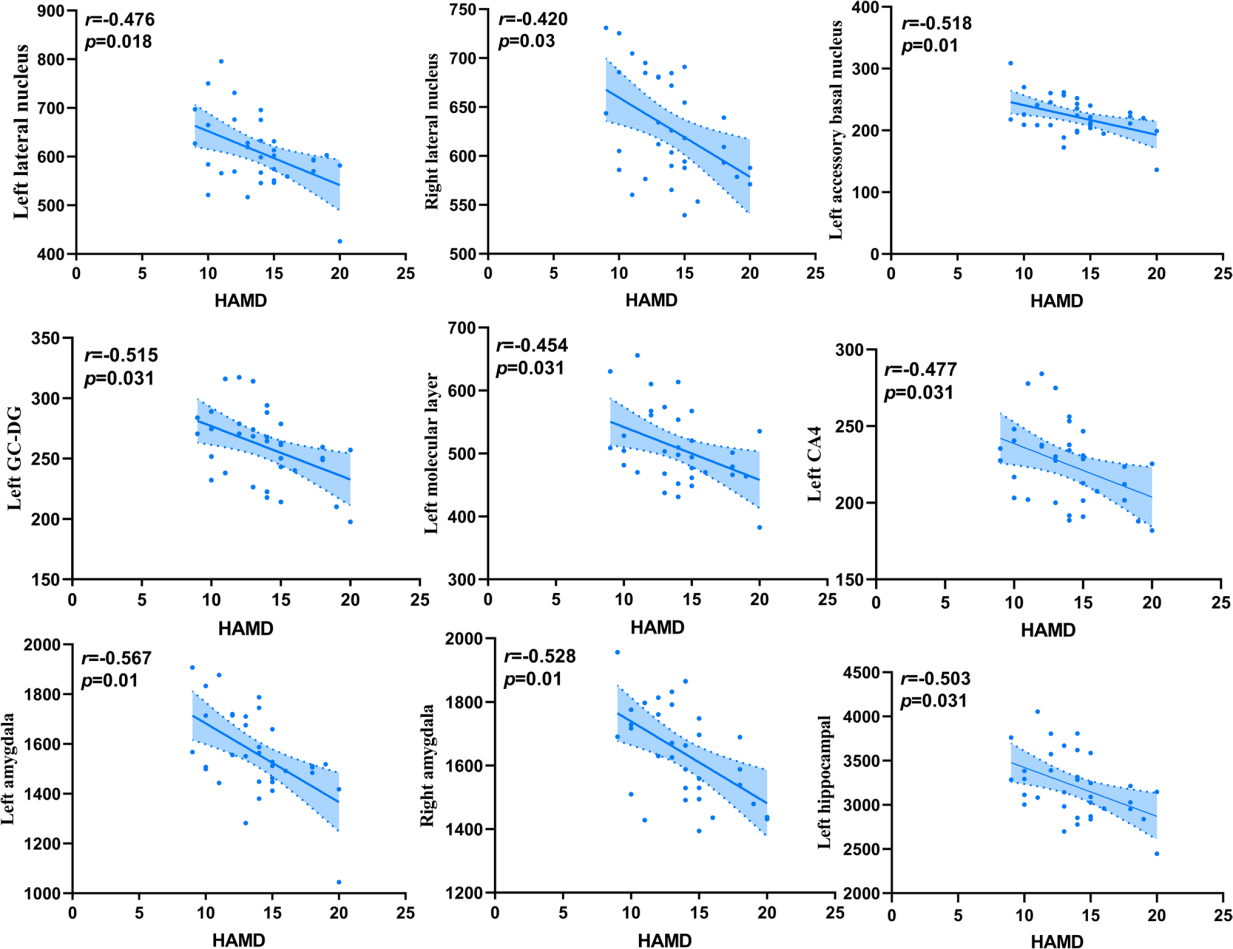
Partial correlation analysis of amygdala and hippocampal subregion volumes with HAMD scores in the DPD group. FDR corrected *P* value < 0.05. Abbreviations: HAMD, Hamilton depression scale; DPD, Depressed PD patients

After FDR correction in the DPD group, partial correlation analysis found that HAMD scores were negatively correlated with the bilateral total amygdala volumes (left *r* = −0.567, *P* = 0.01; right *r* = −0.528,* P* = 0.01), bilateral lateral nuclei (left *r* = −0.476, *P* = 0.018; right *r* = −0.420, *P* = 0.03) and left accessory basal nucleus (*r* = −0.518, *P* = 0.01). Considering the hippocampus, the volumes of left total hippocampal (*r* = −0.503, *P* = 0.031), left GC-DG (*r* = −0.515,* P* = 0.031), left molecular layer (*r* = −0.454, *P* = 0.031) and left CA4 (*r* = −0.477, *P* = 0.031) were negatively correlated with HAMD scores (Fig. 3). There were no significant correlations between disease duration and amygdala and hippocampal subregion volumes in the DPD group (*P* > 0.05).

As a post hoc analysis, we also conducted a hierarchical regression analysis to examine the relationship between HAMA scores and significantly different amygdala and hippocampal subfield volumes in PD patients. None of the results were statistically significant (*P* > 0.05) (Supplementary Table 3–4). In addition, we added HAMA scores as a covariate on the basis of the original covariates (age, gender, education, and eTIV) to exclude the potential impact of anxiety on the results, and analyzed the correlation of significantly reduced amygdala and hippocampal subregion volumes with the severity of depressive symptoms. This result is similar to the correlation analysis result of uncorrected HAMA scores. After FDR correction in the DPD group, HAMD scores were negatively correlated with the bilateral total amygdala volumes (left *r* = -0.567, *P* = 0.01; right *r* =  − 0.530,* P* = 0.01), bilateral lateral nuclei (left *r* =  − 0.476, *P* = 0.02; right *r* =  − 0.422, *P* = 0.033) and left accessory basal nucleus (*r* =  − 0.532, *P* = 0.01). Considering the hippocampus, the volumes of left total hippocampal (*r* =  − 0.491, *P* = 0.043), left GC-DG (*r* =  − 0.496,* P* = 0.043), left molecular layer (*r* =  − 0.455, *P* = 0.043) and left CA4 (*r* =  − 0.454, *P* = 0.043) were negatively correlated with HAMD scores. At the same time, we used the same method to include HAMD scores as a covariate to exclude the potential influence of depression on the results, and analyzed the correlation of subregion volumes with the severity of anxiety symptoms. The results showed no correlation between HAMA scores and amygdala and hippocampal subregion volumes in DPD patients (*P* > 0.05).

### Hippocampal and amygdala subregion volumes and clinical indexes predict DPD

**Fig. 4:**
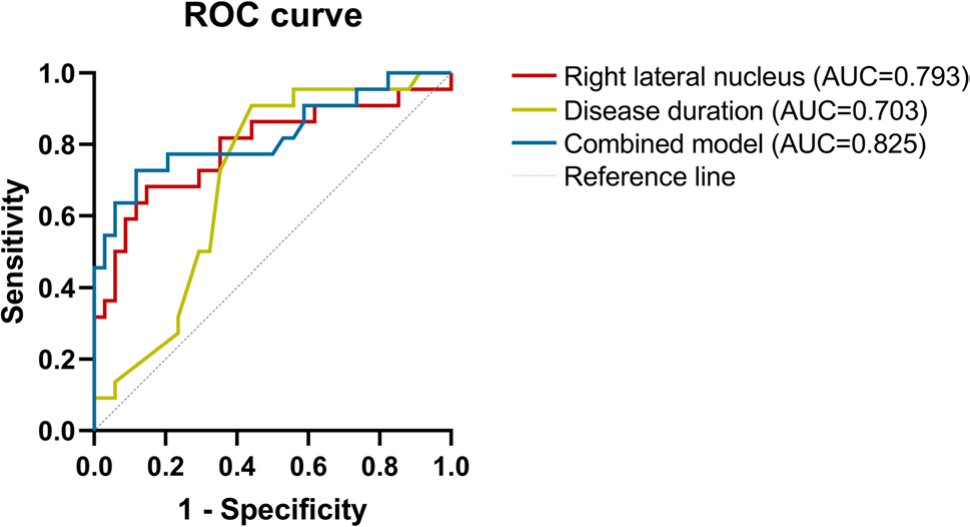
The ROC curves of disease duration, right lateral nucleus volume, and combined model for the discrimination of DPD and NDPD. Abbreviations: DPD, Depressed PD patients; NDPD, Nondepressed PD patients

Multivariable logistic regression analysis showed that the volume of right lateral nuclei (OR = 0.984, *P* = 0.006) and disease duration (OR = 1.267, *P* = 0.033) were independent predictors of DPD (Table 2). At a cutoff of 687.41 mm^3^, the AUC, sensitivity, and specificity of the volume of right lateral nuclei were 0.793, 68.2%, and 85.3%. At a cutoff of 4.5 years, the AUC, sensitivity, and specificity of the disease duration were 0.703, 90.9%, and 55.9%. The combination of the two predictors achieved the best diagnostic performance in distinguishing DPD from NDPD, and the AUC, sensitivity, and specificity were 0.825, 72.7%, and 88.2% (Fig. 4).

**Table 2:** Multiple Logistic regression analysis of DPD patients

	B	SE	OR (95%CI)	*P* value
Right lateral nucleus	−0.016	7.578	0.984 (0.973 ~ 0.995)	0.006
Disease duration	0.237	4.537	1.267 (1.019 ~ 1.576)	0.033

The forward stepwise regression analyses were performed to introduce factors gradually. Disease duration and right lateral nucleus were selected as independent predictors of DPD

Abbreviations: *DPD* Depressed PD patients; *SE* Standard error; *OR* Odd ratio; *CI* Confidence interval

## Discussion

In this study, we analyzed the atrophy patterns of the amygdala and hippocampal subregions and demonstrated an association between partial subregion volumes and the severity of depression in DPD patients. Different subregions may be differently impaired in the pathogenesis of DPD. Moreover, the right lateral nuclei and disease duration were independent predictors of DPD.

Our findings supported prior research that DPD patients had a longer disease duration than NDPD patients (Brown et al., 1988). Depressive symptoms in PD patients usually co-occur with anxiety and interact with each other (Dissanayaka et al., 2014). The present study also demonstrated this, suggesting that depression and anxiety in PD may have similar pathophysiological mechanisms.

Atrophy of the hippocampus and amygdala subregions has been consistently reported in depression. However, little is known about their changes in DPD patients. Our findings help to overcome this gap to a certain extent. Our results showed that DPD group had significantly lower bilateral amygdala volumes than HC and NDPD groups. Moreover, DPD group had a smaller left hippocampus than HC group. However, NDPD group had no hippocampus or amygdala atrophy compared with HC group. Previous studies focused only on the effect of PD pathology on these two regions and did not further involve the influence of depressive disorder (Brück et al., 2004; Harding et al., 2002). As the hippocampus and amygdala are closely related to emotion regulation and processing (Phelps, 2004), we speculate that they also play a crucial role in DPD patients.

Yao et al., (Yao et al., 2020) suggested that the morphological changes in the hippocampus and amygdala subregions may be more significant and sensitive than total volume changes. We showed that DPD patients had atrophy in multiple amygdala subregions, specifically the bilateral lateral nucleus, left accessory basal nucleus, right cortical nucleus, right central nucleus, and right medial nucleus subregions. Unlike our results, Kim et al., (Kim et al., 2021) found only the lateral nucleus and anterior amygdaloid atrophy in major depressive disorder patients. These two studies suggested that the pathology of PD may increase the universality of amygdala subregion atrophy. The lateral nucleus is the primary site receiving inputs from sensory cortices. Consistent with our results, the atrophy of the lateral nucleus may reduce the emotional memory-enhancing effects associated with sensory stimuli and impair emotion regulation (Blair et al., 2001). Harding et al., (Harding et al., 2002) found selective neuronal loss and high LB formation in the cortical and basolateral nucleus. Braak et al., (Braak et al., 1994) revealed that LB and Lewy neurites accumulated primarily in the central and accessory cortical nuclei in PD. The above pathological pattern of the amygdala subregions may promote amygdala neuron loss and volume reduction, disrupt relevant feedforward and feedback connections, and cause depressive symptoms (Braak et al., 1994). The central and medial nuclei are the primary output nuclei of the hypothalamus, and both are particularly sensitive to negative emotional stimuli (Davis & Whalen, 2001). An interesting additional finding of the present study is a decrease in the volume of the right cortical nucleus in DPD group. The cortical nucleus receives significant input from the olfactory bulb (Swanson & Petrovich, 1998), and its atrophy may contribute to the common early dysosmia in PD patients, suggesting that the amygdala subregion is associated with multiple non-motor symptoms in PD.

The present study showed that DPD patients exhibited an asymmetric atrophy pattern in the hippocampus and its subregions, mainly on the left side (the left molecular layer, left GC-DG, left CA3, and left CA4). Previous studies have found a similar asymmetric hippocampal atrophy pattern in patients with major depressive disorder (Frodl et al., 2002; Kronmüller et al., 2009). The neurobiological basis behind this phenomenon has not yet been fully explained, and whether there is a common pathophysiological mechanism between PD and primary depression remains to be further elucidated. At present, the relatively small sample size may limit the statistical power of this study. A larger dataset of participants is necessary to make the results more robust. Roddy et al., (Roddy et al., 2019) showed that hippocampal subregions volume alterations in patients with major depressive disorder are limited to the classical hippocampal subregions, i.e., CA1 to CA4, subiculum, GC-DG, hippocampal tail, and molecular layer; this was consistent with the pattern of hippocampal subregion atrophy in this study. Considering that the classical hippocampal subregion may be the area of the core pathological change of the hippocampus in depression. Györfi et al., (Györfi et al., 2017) found that newly diagnosed PD patients were confined to reduced volume in the CA2 and CA3 subregions. It is possible that the newly diagnosed PD patients had less severe depression, limiting the involvement of the hippocampal subregion compared to our study. The GC-DG and CA subregions are sensitive, and their morphology is the first to be altered under chronic stress (Huang et al., 2013; Stepan et al., 2015). The CA, particularly the pyramidal cells of CA3, is vulnerable to neuronal remodeling and cell loss under chronic stress (Conrad, 2008). Preclinical studies suggest that dendritic retraction in CA3 is more sensitive to chronic psychological stress than in CA1–2 (Czéh & Lucassen, 2007), which may partly explain our findings.

Depression severity negatively correlates with the bilateral amygdala and left hippocampus volume, consistent with previous studies (van Mierlo et al., 2015). More specifically, we found that severe depressive symptoms were correlated with atrophy in multiple amygdala and hippocampal subregions. Our results showed that the volumes of the lateral nucleus and accessory basal nucleus were negatively correlated with the severity of depression. The accessory basal nucleus mainly receives input from the lateral nucleus and orbitofrontal cortex. Its output mainly projects to the central nucleus and striatum, regulating emotional information (Kenwood & Kalin, 2021). Therefore, we speculate that the atrophy of these regions may cause damage to the emotional circuits. Furthermore, we found the right lateral nucleus volume was an independent predictor of DPD. The lateral nuclei is rich in glucocorticoid receptors, rendering it a prime target for stress-induced morphological and molecular alterations (Skórzewska et al., 2015). As the amygdala is a potential neurological biomarker for depression, our study demonstrated the potential value of using the volume of the lateral nuclei of the amygdala in predicting DPD.

Atrophy of the left dentate nucleus, CA4, and molecular layer was negatively correlated with depression severity in DPD patients. Classical tri-synaptic circuit subregions include CA3, CA4, and GC-DG. Disrupting any of these subregions alters circuit function, affecting sensory and emotional information processing and producing depression (Stepan et al., 2015). With chronic stress and glucocorticoid overexposure, the hypothalamic–pituitary–adrenal axis dysregulation and brain-derived neurotrophic factor reduction may affect synaptic maturation of newborn GC-DG neurons and inhibit hippocampus neurogenesis (Czéh & Lucassen, 2007). Low hippocampus volumes may result from decreasing GC-DG neurogenesis (Jacobs et al., 2000). In addition, DPD is also related to hippocampus neurogenesis defects (Lim et al., 2018). The molecular layer contains sensory nerve fibers from the internal olfactory cortex (Amaral et al., 2007); its atrophy may impair subregional connections and emotional information integration, resulting in emotional dysfunctions.

Depressive symptoms in PD patients usually co-occur with anxiety. This study found a moderate correlation between HAMD scores and HAMA scores in PD patients. Therefore, we further exclude the potential impact of anxiety on the results. This result is similar to the correlation analysis result of uncorrected HAMA scores. This may indicate that the correlation between depression scores and amygdala and hippocampal subregion volumes in this study is less affected by anxiety symptoms. In addition, the results of this study show that there is no correlation between HAMA scores and the volume of hippocampus and amygdala subregions, which is inconsistent with Vriend et al. (Vriend et al., 2016). We speculate that different research results may be related to different anxiety scales or research subjects.

There were some limitations in this study: (1) all subjects were recruited from a single hospital, and the sample size was small. In order to verify the reliability of the results of this study, we use a relatively large sample of dataset in the Parkinson Progression Markers Initiative (PPMI) database for replication analysis (http://www.ppmi-info.org), which further proves the reliability and reproducibility of the results of this study. See supplementary material for specific results. (2) the present study did not consider the effects of PD medication and antidepressants in PD patients, which may interfere with the results to a certain extent; (3) this study had limited information on the subject's cognitive status. The MMSE is the most commonly used scale to assess mild cognitive impairment in clinical practice and has about 70% sensitivity for identifying dementia in PD (Hoops et al., 2009). However, cognitive deficits are common, especially in advanced stages, thus, it's possible some advanced PD patients had subtle cognitive impairments that the MMSE failed to identify. In the future a more detailed battery of cognitive assessments in this cohort to enhance our understanding of the cognitive status of these patients.

## Conclusion

This study confirmed that the amygdala and hippocampal subregions are one of the candidate brain regions that might be mostly related to the pathophysiology of DPD. DPD patients showed atrophy in multiple amygdala subregions and left asymmetric hippocampal subregions. In addition, we also found that decreased amygdala and hippocampal subregion volumes were correlated with the severity of depressive symptoms in DPD patients. The right lateral nuclei and disease duration were independent predictors of DPD. Furthermore, a combined model further improved diagnostic performance and may be useful for distinguishing between DPD and NDPD.

### Supplementary Information

Below is the link to the electronic supplementary material.

Supplementary file1 (DOCX 43.7 KB)

## Data Availability

The datasets generated during and/or analysed during the current study are available from the corresponding author on reasonable request.
