# Supplementary material for: Atrophy patterns in hippocampus and amygdala subregions of depressed patients with Parkinson's disease
Source: Brain Imaging Behav. 2024 Jan 3;18(3):475–84. doi: 10.1007/s11682-023-00844-9 (PMC11222218; doi:10.1007/s11682-023-00844-9)
Supplement: Supplementary file 1 — Supplementary file1 (DOCX 43.7 KB) [file 11682_2023_844_MOESM1_ESM.docx]

**Supplementary Table 1.** Intergroup comparison of amygdala subregion volumes

|  | Group | | | ANCOVA | | Post-hoc *p* value | |
| --- | --- | --- | --- | --- | --- | --- | --- |
|  | DPD (n=34) | NDPD (n=22) | HC (n=28) | *P* value | *P*_FDR_ value | DPD vs HC | DPD vs NDPD |
| **Left** |  |  |  |  |  |  |  |
| Whole amygdala | 1560.88±173.54 | 1669.33±137.63 | 1702.39±142.67 | 0.015 | **0.038** | **0.026** | **0.033** |
| Lateral nucleus | 609.55±72.28 | 659.30±60.96 | 675.06±72.42 | 0.010 | **0.029** | **0.016** | 0.056 |
| Basal nucleus | 401.54±48.46 | 421.61±35.75 | 429.85±33.35 | 0.112 | 0.150 | - | - |
| Accessory basal nucleus | 222.39±31.00 | 243.19±22.56 | 250.12±27.50 | 0.008 | **0.029** | **0.012** | 0.059 |
| AAA | 47.36±5.89 | 50.46±6.44 | 51.87±7.64 | 0.097 | 0.139 | - | - |
| Central nucleus | 39.84±10.15 | 42.18±6.55 | 44.10±7.54 | 0.330 | 0.367 | - | - |
| Medial nucleus | 18.64±5.46 | 22.14±5.39 | 20.69±6.36 | 0.094 | 0.139 | - | - |
| Cortical nucleus | 20.01±4.84 | 23.72±3.48 | 23.36±5.65 | 0.058 | 0.102 | - | - |
| CAT | 153.26±16.55 | 158.15±13.54 | 158.78±15.35 | 0.669 | 0.704 | - | - |
| Paralaminar nucleus | 48.31±7.40 | 48.58±5.35 | 48.56±3.84 | 0.987 | 0.987 | - | - |
| **Right** |  |  |  |  |  |  |  |
| Whole amygdala | 1639.69±150.55 | 1795.88±235.07 | 1820.54±183.24 | 0.007 | **0.029** | **0.017** | **0.031** |
| Lateral nucleus | 628.39±53.25 | 703.39±104.04 | 700.19±74.18 | 0.004 | **0.029** | **0.019** | **0.009** |
| Basal nucleus | 420.78±44.07 | 448.27±54.26 | 457.26±44.11 | 0.061 | 0.102 | - | - |
| Accessory basal nucleus | 246.56±30.37 | 267.92±37.08 | 277.29±32.70 | 0.024 | 0.053 | - | - |
| AAA | 51.26±6.88 | 57.14±7.55 | 57.77±11.09 | 0.046 | 0.092 | - | - |
| Central nucleus | 43.50±9.03 | 47.81±7.45 | 52.18±8.61 | 0.005 | **0.029** | **0.004** | 0.380 |
| Medial nucleus | 20.59±6.08 | 24.58±6.23 | 27.07±7.36 | 0.009 | **0.029** | **0.007** | 0.267 |
| Cortical nucleus | 23.56±4.44 | 26.98±5.16 | 28.85±5.10 | 0.004 | **0.029** | **0.004** | 0.104 |
| CAT | 157.64±18.65 | 169.56±27.58 | 170.01±20.38 | 0.171 | 0.214 | - | - |
| Paralaminar nucleus | 47.40±5.08 | 50.23±7.53 | 49.90±5.00 | 0.255 | 0.300 | - | - |

Adjusted age, sex, education, and eTIV.

FDR Correction adjusted *P* value < 0.05 had statistical significance. Bold values indicates statistically significant *P* values. DPD vs HC, DPD vs NDPD.

Abbreviations: DPD, Depressed PD patients; NDPD, Nondepressed PD patients; HC, Healthy controls; AAA, Anterior amygdaloid area; CAT, Cortico-amygdaloid transition area.

**Supplementary Table 2.** Intergroup comparison of hippocampal subregion volumes

|  | Group | | | ANCOVA | | Post-hoc *p* value | |
| --- | --- | --- | --- | --- | --- | --- | --- |
|  | DPD (n=34) | NDPD (n=22) | HC (n=28) | *P* value | *P*_FDR_ value | DPD vs HC | DPD vs NDPD |
| **Left** |  |  |  |  |  |  |  |
| Whole hippocampus | 3210.10±363.80 | 3440.75±390.84 | 3503.35±357.45 | 0.012 | **0.031** | **0.013** | 0.127 |
| Hippocampal tail | 527.81±81.74 | 574.41±85.16 | 549.21±95.76 | 0.532 | 0.575 | - | - |
| Subiculum | 418.44±57.34 | 460.94±56.75 | 459.19±48.38 | 0.017 | 0.089 | - | - |
| CA1 | 580.85±83.17 | 622.56±71.83 | 644.47±87.68 | 0.075 | 0.139 | - | - |
| Hippocampal fissure | 148.49±31.31 | 155.54±31.34 | 145.74±28.26 | 0.579 | 0.579 | - | - |
| Presubiculum | 317.78±41.72 | 333.39±40.18 | 333.41±43.38 | 0.331 | 0.430 | - | - |
| Parasubiculum | 70.04±23.88 | 61.43±12.04 | 60.84±13.39 | 0.127 | 0.206 | - | - |
| Molecular layer | 509.65±60.90 | 552.41±64.19 | 574.78±63.18 | 0.005 | **0.020** | **0.004** | 0.207 |
| GC-DG | 259.85±29.82 | 277.98±36.20 | 291.93±33.74 | 0.004 | **0.020** | **0.003** | 0.411 |
| CA3 | 182.21±25.96 | 191.65±27.97 | 211.79±39.77 | 0.006 | **0.020** | **0.007** | 1.000 |
| CA4 | 225.18±26.29 | 239.00±29.47 | 251.93±27.70 | 0.006 | **0.020** | **0.005** | 0.663 |
| Fimbria | 65.96±23.08 | 75.99±23.76 | 71.20±21.99 | 0.199 | 0.287 | - | - |
| HATA | 52.34±8.23 | 51.01±6.81 | 54.60±9.73 | 0.435 | 0.514 | - | - |
| **Right** |  |  |  |  |  |  |  |
| Whole hippocampal | 3378.17±404.32 | 3683.30±687.70 | 3659.30±355.15 | 0.071 | 0.185 | - | - |
| Hippocampal tail | 566.81±99.53 | 621.86±91.78 | 579.01±88.00 | 0.308 | 0.415 | - | - |
| Subiculm | 419.50±56.70 | 479.55±104.30 | 466.54±43.23 | 0.014 | 0.078 | - | - |
| CA1 | 633.70±80.71 | 682.48±147.99 | 701.41±93.07 | 0.209 | 0.340 | - | - |
| Hippocampal fissure | 169.58±39.63 | 157.71±30.29 | 157.64±33.40 | 0.424 | 0.458 | - | - |
| Presubiculum | 310.21±42.44 | 328.71±64.04 | 306.23±37.64 | 0.319 | 0.415 | - | - |
| Parasubiculum | 63.23±17.78 | 59.85±16.24 | 56.81±12.95 | 0.393 | 0.459 | - | - |
| Molecular layer | 536.18±66.07 | 590.65±122.51 | 602.96±61.77 | 0.041 | 0.133 | - | - |
| GC-DG | 272.51±35.49 | 295.40±63.30 | 313.07±36.16 | 0.014 | 0.078 | - | - |
| CA3 | 204.35±35.14 | 216.63±56.96 | 232.31±32.89 | 0.105 | 0.199 | - | - |
| CA4 | 237.17±32.71 | 255.17±50.76 | 270.54±29.86 | 0.018 | 0.078 | - | - |
| Fimbria | 61.05±22.68 | 69.25±28.58 | 75.85±17.70 | 0.107 | 0.199 | - | - |
| HATA | 53.86±8.64 | 56.25±15.41 | 54.58±8.73 | 0.866 | 0.866 | - | - |

Adjusted with age, sex, education, and eTIV.

FDR Correction adjusted *P* value < 0.05 had statistical significance. Bold values indicate statistically significant *P* values. DPD vs HC.

Abbreviations: DPD, Depressed PD patients; NDPD, Nondepressed PD patients; HC, Healthy controls; GC-DG, Granule cell layer of the dentate gyrus; HATA, Hippocampus-Amygdala transition area.

**Supplementary Table 3.** Hierarchical regression analysis on amygdala volume

|  | Model 1 | | | | Model 2 | | | | | |
| --- | --- | --- | --- | --- | --- | --- | --- | --- | --- | --- |
|  | B | β | P | R2 | B | β | P | R^2^ | ∆R^2^ | Sig |
| **Left Whole amygdala** |  |  |  | 0.16 |  |  |  | 0.18 | 0.10 | 0.22 |
| Constant | 1989.08 | - | <0.001 |  | 2027.76 | - | <0.001 |  |  |  |
| Age | -8.83 | 3.24 | 0.009 |  | -8.50 | -0.34 | 0.010 |  |  |  |
| Gender | -48.31 | -0.15 | 0.28 |  | -32.24 | -0.10 | 0.489 |  |  |  |
| Education | -0.8 | -0.01 | 0.915 |  | -0.82 | -0.01 | -0.913 |  |  |  |
| eTIV | 0.001 | 0.14 | 0.323 |  | 0.001 | 0.13 | 0.361 |  |  |  |
| HAMA |  |  |  |  | -5.29 | -0.17 | 0.221 |  |  |  |
| **Left Lateral nucleus** |  |  |  | 0.15 |  |  |  | 0.19 | 0.11 | 0.10 |
| Constant | 760.88 | - | 0.000 |  | 783.19 | - | 0.000 |  |  |  |
| Age | -2.96 | -0.28 | 0.039 |  | -2.77 | -0.26 | 0.050 |  |  |  |
| Gender | -31.60 | -0.22 | 0.107 |  | -22.34 | -0.16 | 0.262 |  |  |  |
| Education | -0.64 | -0.03 | 0.843 |  | -0.65 | -0.03 | 0.838 |  |  |  |
| eTIV | 0.001 | 0.14 | 0.318 |  | 0.001 | 0.12 | 0.364 |  |  |  |
| HAMA |  |  |  |  | -3.05 | -0.23 | 0.099 |  |  |  |
| **Left Accessory Basal nucleus** |  |  |  | 0.22 |  |  |  | 0.24 | 0.16 | 0.35 |
| Constant | 302.07 | - | 0.000 |  | 307.09 | - | 0.000 |  |  |  |
| Age | -2.02 | -0.46 | 0.001 |  | -1.98 | -0.45 | 0.001 |  |  |  |
| Gender | -3.31 | -0.06 | 0.66 |  | -1.23 | -0.02 | 0.877 |  |  |  |
| Education | 0.47 | 0.05 | 0.714 |  | 0.46 | 0.05 | 0.715 |  |  |  |
| eTIV | 0.001 | 0.17 | 0.200 |  | 0.001 | 0.16 | 0.220 |  |  |  |
| HAMA |  |  |  |  | -0.69 | -0.12 | 0.351 |  |  |  |
| **Right Whole amygdala** |  |  |  | 0.14 |  |  |  | 0.165 | 0.082 | 0.22 |
| Constant | 1993.43 | - | <0.001 |  | 2040.52 | - | <0.001 |  |  |  |
| Age | -6.51 | -0.22 | 0.104 |  | -6.11 | -0.21 | 0.126 |  |  |  |
| Gender | -101.56 | -0.25 | 0.067 |  | -82.0 | -0.21 | 0.151 |  |  |  |
| Education | 7.927 | 0.117 | 0.385 |  | 7.91 | 0.12 | 0.383 |  |  |  |
| eTIV | 0.001 | 0.09 | 0.540 |  | 0.001 | 0.07 | 0.593 |  |  |  |
| HAMA |  |  |  |  | -6.44 | -0.17 | 0.220 |  |  |  |
| **Right Lateral nucleus** |  |  |  | 0.18 |  |  |  | 0.22 | 0.14 | 0.10 |
| Constant | 859.13 | - | 0.000 |  | 885.16 | - | 0.000 |  |  |  |
| Age | -3.12 | -0.25 | 0.060 |  | -2.90 | -0.23 | 0.076 |  |  |  |
| Gender | -48.87 | -0.29 | 0.033 |  | -38.06 | -0.23 | 0.102 |  |  |  |
| Education | 4.02 | 0.14 | 0.285 |  | 4.01 | 0.14 | 0.278 |  |  |  |
| eTIV | 0.001 | 0.028 | 0.837 |  | 0.001 | 0.013 | 0.921 |  |  |  |
| HAMA |  |  |  |  | -3.56 | -0.22 | 0.098 |  |  |  |
| **Right Central nucleus** |  |  |  | 0.07 |  |  |  | 0.08 | -0.01 | 0.44 |
| Constant | 53.37 | - | 0.004 |  | 54.71 | - | 0.004 |  |  |  |
| Age | -0.04 | -0.03 | 0.805 |  | -0.03 | -0.03 | 0.856 |  |  |  |
| Gender | -4.19 | -0.24 | 0.09 |  | -3.63 | -0.21 | 0.158 |  |  |  |
| Education | 0.17 | 0.06 | 0.681 |  | 0.17 | 0.06 | 0.684 |  |  |  |
| eTIV | 0.001 | -0.01 | 0.953 |  | 0.001 | -0.02 | 0.913 |  |  |  |
| HAMA |  |  |  |  | -0.18 | -0.11 | 0.436 |  |  |  |
| **Right Medial nucleus** |  |  |  | 0.04 |  |  |  | 0.07 | -0.02 | 0.25 |
| Constant | 39.00 | - | 0.05 |  | 40.49 | - | 0.004 |  |  |  |
| Age | -0.03 | -0.03 | 0.816 |  | -0.02 | -0.02 | 0.891 |  |  |  |
| Gender | -2.31 | -0.18 | 0.209 |  | -1.69 | -0.13 | 0.373 |  |  |  |
| Education | -0.13 | -0.06 | 0.678 |  | -0.13 | -0.06 | 0.675 |  |  |  |
| eTIV | 0.001 | -0.15 | 0.323 |  | 0.001 | -0.16 | 0.288 |  |  |  |
| HAMA |  |  |  |  | -0.20 | -0.17 | 0.247 |  |  |  |
| **Right Cortical nucleus** |  |  |  | 0.08 |  |  |  | 0.11 | -0.02 | 0.22 |
| Constant | 32.05 | - | 0.003 |  | 33.26 | - | 0.002 |  |  |  |
| Age | -0.15 | -0.20 | 0.147 |  | -0.14 | -0.19 | 0.176 |  |  |  |
| Gender | -1.52 | -0.15 | 0.280 |  | -1.02 | -0.10 | 0.483 |  |  |  |
| Education | -0.10 | -006 | 0.659 |  | -0.10 | -0.06 | 0.655 |  |  |  |
| eTIV | 0.001 | 0.10 | 0.478 |  | 0.001 | 0.09 | 0.528 |  |  |  |
| HAMA |  |  |  |  | -0.17 | -0.18 | 0.220 |  |  |  |

**Supplementary Table 4.** Hierarchical regression analysis on hippocampal volume

|  | Model 1 | | | | Model 2 | | | | | |
| --- | --- | --- | --- | --- | --- | --- | --- | --- | --- | --- |
|  | B | β | P | R2 | B | β | P | R^2^ | ∆R^2^ | Sig |
| **Right whole hippocampus** |  |  |  | 0.16 |  |  |  | 0.18 | 0.10 | 0.28 |
| Constant | 4174.19 | - | 0.000 |  | 4252.98 | - | 0.000 |  |  |  |
| Age | -21.17 | -0.37 | 0.007 |  | -20.50 | -0.36 | 0.009 |  |  |  |
| Gender | -92.08 | -0.12 | 0.376 |  | -59.35 | -0.08 | 0.583 |  |  |  |
| Education | 11.21 | 0.09 | 0.518 |  | 11.17 | 0.09 | 0.518 |  |  |  |
| eTIV | 0.001 | 0.11 | 0.416 |  | 0.001 | 0.10 | 0.457 |  |  |  |
| HAMA |  |  |  |  | -10.78 | -0.15 | 0.281 |  |  |  |
| **Left Molecular layer** |  |  |  | 0.20 |  |  |  | 0.23 | 0.15 | 0.22 |
| Constant | 677.76 | - | 0.000 |  | 692.50 | - | 0.000 |  |  |  |
| Age | -3.82 | -0.40 | 0.003 |  | -3.69 | -0.39 | 0.004 |  |  |  |
| Gender | -20.34 | -0.16 | 0.003 |  | -14.22 | -0.11 | 0.420 |  |  |  |
| Education | 2.81 | 0.13 | 0.323 |  | 2.80 | 0.13 | 0.322 |  |  |  |
| eTIV | 0.001 | 0.125 | 0.350 |  | 0.001 | 0.11 | 0.391 |  |  |  |
| HAMA |  |  |  |  |  |  |  |  |  |  |
| **Left GC-DG** |  |  |  | 0.11 |  |  |  | 0.14 | 0.05 | 0.22 |
| Constant | 294.58 | - | 0.000 |  | 302.56 | - | 0.000 |  |  |  |
| Age | -1.17 | -0.24 | 0.082 |  | -1.11 | -0.23 | 0.48 |  |  |  |
| Gender | -9.99 | -0.15 | 0.279 |  | -6.67 | -0.10 | 0.484 |  |  |  |
| Education | 1.57 | 0.14 | 0.308 |  | 1.56 | 0.14 | 0.306 |  |  |  |
| eTIV | 0.001 | 0.12 | 0.385 |  | 0.001 | 0.11 | 0.429 |  |  |  |
| HAMA |  |  |  |  | -1.09 | -0.17 | 0.216 |  |  |  |
| **Left CA4** |  |  |  | 0.12 |  |  |  | 0.15 | 0.06 | 0.23 |
| Constant | 234.68 | - | 0.000 |  | 241.18 | - | 0.000 |  |  |  |
| Age | -0.89 | -0.21 | 0.118 |  | -0.83 | -0.20 | 0.142 |  |  |  |
| Gender | -9.60 | -0.17 | 0.215 |  | -6.90 | -0.12 | 0.389 |  |  |  |
| Education | 1.50 | 0.16 | 0.25 |  | 1.49 | 0.16 | 0.245 |  |  |  |
| eTIV | 0.001 | 0.16 | 0.25 |  | 0.001 | 0.15 | 0.278 |  |  |  |
| HAMA |  |  |  |  | -0.89 | -0.17 | 0.229 |  |  |  |

**Supplementary results**

To verify the reliability of the results of this study, we include 125 PD patients (65 DPD and 60 NDPD) and 37 HCs from the Parkinson Progression Markers Initiative (PPMI) database (http://www.ppmi-info.org). We used the same statistical method and multiple comparison corrections to compare the volume differences of hippocampus and amygdala among the three groups. The results are as follows:

**Demographic and clinical data**

The demographic information and clinical data are summarized in **Supplementary Table 5.** The three groups had similar gender, age, education, H&Y stage and MOCA results (*P* > 0.05). Geriatric Depression Scale-Short (GDS-15) scores were significantly higher in DPD group than NDPD group (*P* < 0.01).

**Amygdala subregion volume differences among DPD, NDPD, and HC groups**

The bilateral global amygdala volumes were significantly lower in DPD group than in NDPD group (left *P* = 0.004, right *P* = 0.007) and HC group (left *P* = 0.028, right *P* = 0.001). Further comparison of bilateral amygdala subregion volumes revealed significantly lower volumes in the bilateral lateral nucleus, bilateral accessory basal nucleus, bilateral AAA, right basal nucleus, right cortical nucleus and right central nucleus among the three groups (all *P* < 0.05). Post-hoc analyses showed significantly lower volumes in the DPD group than in the HC group and NDPD group for the bilateral lateral nuclei, bilateral accessory basal nucleus, right AAA and right basal nucleus. Moreover, the right cortical nucleus and right central nucleus volumes were significantly lower in DPD and NDPD groups than in HC group. compared with NDPD group, DPD group showed a significantly lower volume in the left AAA. (**Supplementary Table 6**).

**Hippocampal subregion volume differences among DPD, NDPD, and HC groups**

The DPD group's bilateral global hippocampal volume was significantly reduced than in HC group (left *P* = 0.001, right *P* < 0.001) and NDPD group (left *P* = 0.007, right *P* = 0.002). Further comparison of hippocampal subregion volumes revealed significantly lower volumes in the bilateral subiculum, bilateral molecular layer, bilateral GC-DG, bilateral CA1, left CA3 and bilateral CA4 among the three groups (all *P <* 0.05). Post-hoc analyses showed that all 10 hippocampal subregions except the left CA3 in DPD group had a significantly lower volume compared with NDPD group and HC group. In the DPD group, the left CA3 volume is only smaller than the HC group. Moreover, the volumes of right subiculum, right GC-DG, right CA4 and right molecular layer in NDPD group were significantly smaller than those in HC group (**Supplementary Table 7**).

Our results are generally consistent and replicable with the research on relatively large sample PPMI dataset, although the PPMI dataset shows more subregions with significant differences. In our results, there were significant differences in the volumes of right accessory basal nucleus, right AAA, bilateral subiculum, right molecular layer, right GC-DG and right CA4 among the three groups before FDR correction. This is similar to the result of the PPMI database. Unfortunately, these results did not survive correction for multiple comparisons. The relatively small sample size of patients may limit the statistical power of this study. Other slightly inconsistent results may be due to the heterogeneity (eg, age, sex, education, disease severity), race and sample size of patients, and different depression scales may have different sensitivity and evaluation criteria in distinguishing patients with DPD and NDPD. In conclusion, the results of PPMI database further verify the reliability of our results.

**Supplementary Table 5.** Demographic and clinical characteristics

| Characteristics | DPD (n=65) | NDPD (n=60) | HC (n=37) | χ^2^/*F*/*t/Z* | *P* value |
| --- | --- | --- | --- | --- | --- |
| Age (years) | 64.94±5.63 | 62.92±7.00 | 63.57±7.59 | 1.502 | 0.226 |
| Gender (male/female) | 41/28 | 32/27 | 20/17 | 0.451 | 0.798 |
| Education (years) | 15.18±3.57 | 16.12±2.68 | 15.35±1.89 | 1.708 | 0.185 |
| H&Y stage (in %) |  |  | - | -0.284 | 0.776 |
| 1 | 27(42%) | 26(43%) |  |  |  |
| 2 | 36(55%) | 33(55%) |  |  |  |
| 3 | 2(3%) | 1(2%) |  |  |  |
| MOCA | 26.55±2.09 | 27.22±1.83 | 27.08±1.64 | 2.061 | 0.131 |
| GDS-15 | 9.95±1.81 | 1.75±1.61 |  | 26.71 | **< 0.001** |

Chi-Squared test, Analysis of variance (ANOVA), Two-sample *t*-test, Mann-Whitney *U* test.

*P* < 0.05 had statistical significance. Bold values indicates statistically significant *P* values. Abbreviations: DPD, Depressed PD patients; NDPD, Nondepressed PD patients; HC, Healthy controls; H&Y, Hoehn and Yahr stage; MoCA, Montreal Cognitive Assessment; GDS-15, Geriatric Depression Scale-Short.

**Supplementary Table 6.** Intergroup comparison of amygdala subregion volumes

|  | Group | | | ANCOVA | | Post-hoc *p* value | | |
| --- | --- | --- | --- | --- | --- | --- | --- | --- |
|  | DPD  (n=65) | NDPD  (n=60) | HC  (n=37) | *P*  value | *P*_FDR_  value | DPD  vs HC | DPD  vs NDPD | NDPD  vs HC |
| **Left** |  |  |  |  |  |  |  |  |
| Whole amygdala | 1669.43±151.96 | 1766.54±220.46 | 1761.17±130.89 | 0.008 | **0.018** | **0.028** | **0.004** | 0.728 |
| Lateral nucleus | 632.29±59.87 | 666.32±79.99 | 680.80±64.65 | 0.002 | **0.006** | **0.001** | **0.005** | 0.416 |
| Basal nucleus | 425.26±44.02 | 443.09±55.22 | 441.87±45.30 | 0.158 | 0.176 | - | **-** | **-** |
| Accessory basal nucleus | 245.74±26.55 | 263.87±42.06 | 262.33±23.55 | 0.013 | **0.024** | **0.024** | **0.007** | 0.934 |
| AAA | 50.39±5.83 | 54.92±8.23 | 52.67±7.11 | 0.010 | **0.020** | 0.238 | **0.003** | 0.143 |
| Central nucleus | 46.78±8.33 | 47.82±9.67 | 44.16±7.14 | 0.112 | 0.132 | - | - | - |
| Medial nucleus | 22.90±6.41 | 23.43±7.25 | 20.56±5.92 | 0.078 | 0.100 | - | - | - |
| Cortical nucleus | 24.99±4.02 | 26.45±5.84 | 25.32±5.03 | 0.080 | 0.100 | - | - | - |
| CAT | 169.76±16.77 | 170.98±17.25 | 163.08±15.49 | 0.073 | 0.100 | - | - | - |
| Paralaminar nucleus | 49.62±5.51 | 51.16±6.54 | 49.46±5.14 | 0.177 | 0.186 | - | - | - |
| **Right** |  |  |  |  |  |  |  |  |
| Whole amygdala | 1714.65±169.09 | 1805.64±186.08 | 1844.72±148.79 | 0.001 | **0.005** | **0.001** | **0.007** | 0.294 |
| Lateral nucleus | 647.20±66.10 | 676.38±66.88 | 704.82±61.44 | 0.000 | **0.000** | **0.000** | **0.022** | **0.036** |
| Basal nucleus | 433.51±45.55 | 458.52±50.45 | 464.38±38.11 | 0.002 | **0.006** | **0.003** | **0.004** | 0.626 |
| Accessory basal nucleus | 260.01±29.74 | 276.72±36.47 | 283.57±28.70 | 0.003 | **0.008** | **0.001** | **0.013** | 0.272 |
| AAA | 53.62±6.49 | 57.34±8.28 | 60.40±9.89 | 0.001 | **0.005** | **0.000** | **0.034** | 0.064 |
| Central nucleus | 47.10±7.74 | 50.67±9.78 | 54.89±10.02 | 0.002 | **0.006** | **0.000** | 0.085 | **0.044** |
| Medial nucleus | 23.71±5.70 | 25.04±8.06 | 26.66±6.74 | 0.352 | 0.352 | - | - | - |
| Cortical nucleus | 26.54±4.11 | 28.07±4.83 | 31.72±8.44 | 0.000 | **0.000** | **0.000** | 0.380 | **0.001** |
| CAT | 173.36±2.38 | 181.97±21.92 | 173.64±18.65 | 0.073 | 0.100 | - | - | - |
| Paralaminar nucleus | 49.60±5.51 | 51.43±6.16 | 50.38±5.58 | 0.077 | 0.100 | - | - | - |

Adjusted age, sex, education, and eTIV.

FDR Correction adjusted *P* value < 0.05 had statistical significance. Bold values indicates statistically significant *P* values. DPD vs HC, DPD vs NDPD.

Abbreviations: DPD, Depressed PD patients; NDPD, Nondepressed PD patients; HC, Healthy controls; AAA, Anterior amygdaloid area; CAT, Cortico-amygdaloid transition area.

**Supplementary Table 7.** Intergroup comparison of hippocampal subregion volumes

|  | Group | | | ANCOVA | | Post-hoc *p* value | | |
| --- | --- | --- | --- | --- | --- | --- | --- | --- |
|  | DPD  (n=65) | NDPD  (n=60) | HC  (n=37) | *P*  value | *P*_FDR_  value | DPD  vs HC | DPD  vs NDPD | NDPD  vs HC |
| **Left** |  |  |  |  |  |  |  |  |
| Whole hippocampus | 3319.04±317.88 | 3516.19±407.62 | 3570.96±303.50 | 0.002 | **0.005** | **0.001** | **0.007** | 0.396 |
| Hippocampal tail | 535.79±78.97 | 566.70±91.28 | 579.27±81.11 | 0.045 | 0.084 | - | - | - |
| Subiculum | 425.52±40.95 | 450.45±54.94 | 462.61±45.33 | 0.001 | **0.003** | **0.000** | **0.003** | 0.312 |
| CA1 | 614.11±65.97 | 655.27±92.37 | 654.72±75.52 | 0.018 | **0.036** | **0.025** | **0.012** | 0.957 |
| Hippocampal fissure | 159.26±23.45 | 162.30±33.67 | 152.25±24.40 | 0.150 | 0.186 | - | - | - |
| Presubiculum | 313.61±35.28 | 324.92±41.42 | 330.72±38.81 | 0.107 | 0.140 | - | - | - |
| Parasubiculum | 71.64±15.49 | 71.49±16.76 | 69.22±16.00 | 0.472 | 0.491 | **-** | - | **-** |
| Molecular layer | 532.92±52.45 | 566.39±70.15 | 582.21±55.44 | 0.001 | **0.003** | **0.000** | **0.008** | 0.162 |
| GC-DG | 268.45±28.06 | 287.74±37.41 | 298.92±29.81 | 0.000 | **0.000** | **0.000** | **0.007** | 0.065 |
| CA3 | 199.11±25.62 | 210.83±37.41 | 220.91±29.06 | 0.009 | **0.020** | **0.002** | 0.117 | 0.090 |
| CA4 | 233.88±24.73 | 248.30±32.01 | 257.87±25.14 | 0.001 | **0.003** | **0.000** | **0.021** | 0.079 |
| Fimbria | 67.66±20.32 | 72.96±17.18 | 74.48±19.47 | 0.381 | 0.413 | - | - | - |
| HATA | 56.35±8.30 | 60.58±10.15 | 57.13±8.56 | 0.070 | 0.107 | - | - | - |
| **Right** |  |  |  |  |  |  |  |  |
| Whole hippocampal | 3417.45±332.98 | 3634.35±385.89 | 3719.82±307.36 | 0.000 | **0.000** | **0.000** | **0.002** | 0.236 |
| Hippocampal tail | 570.72±78.65 | 603.42±86.69 | 600.19±90.63 | 0.108 | 0.140 | - | - | - |
| Subiculm | 424.88±41.61 | 445.93±52.90 | 470.52±38.04 | 0.000 | **0.000** | **0.000** | **0.012** | **0.013** |
| CA1 | 641.07±73.77 | 692.49±85.60 | 713.89±79.53 | 0.000 | **0.000** | **0.000** | **0.002** | 0.176 |
| Hippocampal fissure | 161.71±22.97 | 165.93±31.66 | 154.20±35.68 | 0.052 | 0.090 | - | - | - |
| Presubiculum | 291.11±3.01 | 304.77±36.67 | 306.37±34.62 | 0.066 | 0.101 | - | - | - |
| Parasubiculum | 64.99±13.09 | 67.11±10.73 | 62.84±13.86 | 0.160 | 0.189 | - | - | - |
| Molecular layer | 547.86±56.51 | 586.07±66.96 | 614.43±56.00 | 0.000 | **0.000** | **0.000** | **0.002** | **0.021** |
| GC-DG | 283.80±29.97 | 302.45±35.99 | 320.90±32.44 | 0.000 | **0.000** | **0.000** | **0.005** | **0.009** |
| CA3 | 222.30±29.70 | 235.78±36.57 | 236.87±31.36 | 0.102 | 0.140 | - | - | - |
| CA4 | 245.83±26.33 | 261.08±31.80 | 277.04±27.28 | 0.000 | **0.000** | **0.000** | **0.007** | **0.011** |
| Fimbria | 63.98±18.23 | 69.24±21.80 | 71.80±15.78 | 0.228 | 0.258 | - | - | - |
| HATA | 60.90±9.11 | 63.34±8.91 | 61.74±10.40 | 0.521 | 0.521 | - | - | - |

Adjusted with age, sex, education, and eTIV.

FDR Correction adjusted *P* value < 0.05 had statistical significance. Bold values indicate statistically significant *P* values. DPD vs HC.

Abbreviations: DPD, Depressed PD patients; NDPD, Nondepressed PD patients; HC, Healthy controls; GC-DG, Granule cell layer of the dentate gyrus; HATA, Hippocampus-Amygdala transition area.
